# Social Determinants of Sleep Health Inequities Among Rural Appalachian Adults

**DOI:** 10.1001/jamanetworkopen.2026.5908

**Published:** 2026-04-09

**Authors:** Mairead E. Moloney, Emily Slade, Joon Chung, Maliha Mehnaz Mitu, Michael A. Grandner, Daniela C. Moga

**Affiliations:** 1 Department of Informatics and Health Data Science, Miller School of Medicine, University of Miami, Miami, Florida; 2 Frost Institute for Data Science and Computing, University of Miami, Miami, Florida; 3 Department of Biostatistics, College of Public Health, University of Kentucky, Lexington; 4 Sleep and Health Research Program, Department of Psychiatry, University of Arizona College of Medicine, Tucson; 5 Department of Pharmacy Science and Practice, College of Pharmacy, University of Kentucky, Lexington; 6 Department of Epidemiology and Environmental Health, College of Public Health, University of Kentucky

## Abstract

This cross-sectional study evaluates the association of sociodemographic, health behavior, and psychosocial factors with insufficient sleep duration, insomnia, and obstructive sleep apnea risk among rural Appalachian adults.

## Introduction

Sleep deficiencies—including insufficient sleep duration, poor sleep quality, and clinical sleep disorders such as insomnia and obstructive sleep apnea (OSA)—represent a significant public health burden, particularly among populations experiencing health inequities.^1,2^ Rural Appalachian communities include a federally designated health disparity population with documented geographic insufficient sleep hotspots, yet the prevalence of clinical sleep disorders and their social determinants remain poorly characterized.^3,4,5^ This study investigates the sociodemographic, health behavior, and psychosocial associations of 3 sleep outcomes (insufficient sleep duration, insomnia, and obstructive sleep apnea risk) among rural Appalachian adults.

## Methods

This study followed the Strengthening the Reporting of Observational Studies in Epidemiology (STROBE) reporting guideline for cross-sectional studies. Procedures were approved by the University of Kentucky institutional review board and participants provided informed consent electronically. We analyzed cross-sectional baseline survey data (2023-2025) from the Researching Equitable Sleep Time in Kentucky Communities (REST-KY) study^6^ conducted across 12 economically distressed Eastern Kentucky counties. Eligible participants were English-proficient adults, aged 18 years or older, with internet access and no relocation plans within 24 months. Participants were recruited through print and broadcast media and community venues using convenience sampling. Primary outcomes were clinically significant insomnia (Insomnia Severity Index score ≥10), elevated OSA risk (a STOP-Bang score of ≥3, measured by the following: snoring, tiredness, observed apnea, pressure, body mass index >35 [calculated as weight in kilograms divided by height in meters squared], age >50 years, neck circumference >16 in or 40 cm, and gender), and insufficient sleep duration (<7 hours per night). Participants self-reported independent variable data including sociodemographic factors (age, sex, race, ethnicity, education, employment status, income, and living alone or with others), health indicators and behaviors (alcohol use, smoking, diet quality, physical activity, body mass index, overall health, polypharmacy [≥5 prescription medications], and use of sleep medications), and psychosocial factors (trauma, stress, anxiety or depression, and social support) via well-validated scales (eAppendix in Supplement 1). We examined the bivariate association between each sleep outcome and participant characteristics using Fisher exact tests. All analyses were conducted using R version 4.2.0 (R Project for Statistical Computing) with a 2-sided *P* < .05 considered statistically significant.

## Results

A total of 327 participants had a mean (SD) age of 45.3 (13.1) years and were predominantly female (247 participants [75.5%]). A total of 11 participants (3.6%) reported Hispanic, Latin(a/o), or Latinx ethnicity; 296 (96.4%) reported non-Hispanic, non-Latin(a/o), or non-Latinx ethnicity; and 313 (97.2%) reported White race. Most (288 participants [88.1%]) had complete insomnia data, 269 (82.3%) had complete OSA data, and 281 (85.9%) had complete insufficient sleep data. (Table 1). Among 288 participants, 187 (64.9%) had clinically significant insomnia, 138 of 269 (51.3%) had elevated OSA risk, and 126 of 281 (44.8%) had insufficient sleep. Insomnia prevalence varied significantly by income, declining from 82.9% (34 of 41 participants) among participants earning less than $20 000 annually to 44.4% (16 of 36 participants) among those earning more than $100 000, demonstrating a clear socioeconomic gradient. Insomnia was significantly associated with female sex, non–full-time employment, living alone, cigarette use, poor diet quality, lower social support, poor self-rated health, polypharmacy, trauma history, moderate to severe anxiety or depression, and high stress. Elevated OSA risk was significantly associated with older age, being male, cigarette use, higher body mass index, poor self-rated health, polypharmacy, and trauma history. Insufficient sleep was significantly associated with lower social support (Table 2).

**Table 1.** Participant Characteristics (N = 327)

Characteristic	Participants, No. (%)^a^
Age, y	
18-44	149 (46.0)
45-64	154 (47.5)
≥65	21 (6.5)
Unknown	3
Sex	
Female	247 (75.5)
Male	80 (24.5)
Race	
African American or Black	NA
White	313 (97.2)
Other^b^	NA
Unknown	5
Ethnicity	
Hispanic, Latin(a/o), or Latinx	11 (3.6)
Non-Hispanic, non-Latin(a/o), or non-Latinx	296 (96.4)
Unknown	20
Education	
High school, GED, or less	64 (19.6)
Some college or completed trade school	75 (23.0)
Associate’s degree	61 (18.7)
Bachelor’s degree	60 (18.4)
Graduate or professional school	66 (20.2)
Unknown	1
Employment status	
Employed full-time	205 (63.3)
Employed part-time	30 (9.3)
Retired	30 (9.3)
Unemployed, disability, full-time student, or homemaker	59 (18.2)
Unknown	3
Income, US $	
<20 000	53 (16.5)
20 000-39 999	77 (24.0)
40 000-59 999	64 (19.9)
60 000-79 999	48 (15.0)
80 000-99 999	40 (12.5)
≥100 000	39 (12.1)
Unknown	6
Living alone	
No	255 (79.4)
Yes	66 (20.6)
Unknown	6
Heavy drinking	
No	255 (91.4)
Yes	24 (8.6)
Unknown	48
Cigarette use in past mo	
No	250 (89.0)
Yes	31 (11.0)
Unknown	46
Diet quality	
Less healthy diet	243 (89.7)
More healthy diet	28 (10.3)
Unknown	56
Moderate to vigorous physical activity	
No	157 (56.3)
Yes	122 (43.7)
Unknown	48
Social support score	
0-49.9	46 (15.8)
50-74.9	68 (23.4)
75-100	177 (60.8)
Unknown	36
Body mass index category^c^	
Underweight or normal weight	56 (19.2)
Overweight	75 (25.7)
Obese	161 (55.1)
Unknown	35
Overall health	
Excellent or very good	96 (32.3)
Good	128 (43.1)
Fair or poor	73 (24.6)
Unknown	30
Polypharmacy (≥5 prescription medications)	
No	190 (71.2)
Yes	77 (28.8)
Unknown	60
Use of sleep medications	
No	223 (74.8)
Yes	75 (25.2)
Unknown	29
Trauma	
No	90 (32.7)
Yes	185 (67.3)
Unknown	52
Anxiety or depression	
No	171 (62.9)
Yes	101 (37.1)
Unknown	55
Moderate to high stress	
No	107 (37.9)
Yes	175 (62.1)
Unknown	45

Abbreviation: NA, not available.

^a^ Proportions representing less than 5 people, indicated by NA, were suppressed to protect participant anonymity. Demographics were self-reported by participants.

^b^ The racial category of other includes participants with any of the following responses: American Arab, Middle Eastern, and North African; American Indian or Alaska Native; Asian; Native American or Pacific Islander; biracial or multiracial; or not listed.

^c^ Body mass index calculated as weight in kilograms divided by height in meters squared.

**Table 2.** Prevalence of Sleep Outcomes by Participant Characteristics

Characteristic	Participants, No./total No. (%)^a^		
	Insomnia	Elevated OSA risk	Insufficient sleep
Age, y			
18-44	85/129 (65.9)	43/115 (37.4)	49/123 (39.8)
45-64	91/140 (65.0)	81/135 (60.0)	69/138 (50.0)
≥65	11/19 (57.9)	14/19 (73.7)	8/20 (40.0)
* P* value	.79	<.001	.24
Sex			
Female	155/223 (69.5)	93/210 (44.3)	96/217 (44.2)
Male	32/65 (49.2)	45/59 (76.3)	30/64 (46.9)
* P* value	.003	<.001	.78
Race			
African American or Black	NA	NA	NA
White	181/277 (65.3)	133/258 (51.6)	119/270 (44.1)
Other^b^	NA	NA	NA
* P* value	.82	>.99	.11
Ethnicity			
Hispanic, Latin(a/o), or Latinx	NA	NA	NA
Non-Hispanic, non-Latin(a/o), or non-Latinx	NA	NA	NA
* P* value	>.99	.49	.47
Education			
High school, GED, or less	41/57 (71.9)	30/52 (57.7)	24/55 (43.6)
Some college or completed trade school	47/63 (74.6)	36/58 (62.1)	24/57 (42.1)
Associate’s degree	34/53 (64.2)	24/49 (49.0)	27/53 (50.9)
Bachelor’s degree	30/52 (57.7)	18/50 (36.0)	21/53 (39.6)
Graduate or professional school	34/62 (54.8)	29/59 (49.2)	30/62 (48.4)
* P* value	.10	.08	.75
Employment status			
Employed full time	107/181 (59.1)	77/167 (46.1)	84/176 (47.7)
Employed part time	17/26 (65.4)	12/26 (46.2)	10/26 (38.5)
Retired	18/28 (64.3)	19/27 (70.4)	14/28 (50.0)
Unemployed, disability, full-time student, or homemaker	42/50 (84.0)	27/46 (58.7)	18/48 (37.5)
* P* value	.009	.08	.51
Income, US $			
<20 000	34/41 (82.9)	21/38 (55.3)	15/40 (37.5)
20 000-39 999	54/70 (77.1)	38/64 (59.4)	33/68 (48.5)
40 000-59 999	40/59 (67.8)	26/54 (48.1)	25/57 (43.9)
60 000-79 999	18/42 (42.9)	22/40 (55.0)	19/41 (46.3)
80 000-99 999	22/35 (62.9)	10/32 (31.3)	17/34 (50.0)
≥100 000	16/36 (44.4)	17/36 (47.2)	15/36 (41.7)
* P* value	<.001	.18	.87
Living alone			
No	136/224 (60.7)	104/210 (49.5)	92/219 (42.0)
Yes	47/58 (81.0)	32/54 (59.3)	30/56 (53.6)
* P* value	.003	.22	.13
Heavy drinking			
No	NA	121/237 (51.1)	108/242 (44.6)
Yes	NA	12/23 (52.2)	13/24 (54.2)
* P* value	.11	>.99	.40
Cigarette use in past month			
No	157/245 (64.1)	111/233 (47.6)	108/239 (45.2)
Yes	25/30 (83.3)	22/28 (78.6)	14/29 (48.3)
* P* value	.04	.002	.84
Diet quality			
Less healthy diet	165/237 (69.6)	122/226 (54.0)	110/232 (47.4)
Healthier diet	12/28 (42.9)	9/26 (34.6)	12/27 (44.4)
* P* value	.01	.07	.84
Moderate to vigorous physical activity			
No	104/154 (67.5)	76/146 (52.1)	68/151 (45.0)
Yes	76/120 (63.3)	57/114 (50.0)	55/115 (47.8)
* P* value	.52	.80	.71
Social support score			
0-49.9	36/45 (80.0)	23/40 (57.5)	28/44 (63.6)
50-74.9	51/66 (77.3)	34/62 (54.8)	30/64 (46.9)
75-100	99/174 (56.9)	80/166 (48.2)	68/170 (40.0)
* P* value	<.001	.49	.02
Body mass index^c^			
≤24.9, Underweight or normal weight	33/56 (58.9)	14/53 (26.4)	26/53 (49.1)
25.0-29.9, Overweight	51/71 (71.8)	25/69 (36.2)	31/70 (44.3)
≥30.0, Obese	100/157 (63.7)	99/147 (67.3)	68/154 (44.2)
* P* value	.30	<.001	.82
Overall health			
Excellent or very good	49/95 (51.6)	31/90 (34.4)	37/92 (40.2)
Good	82/121 (67.8)	55/110 (50.0)	54/119 (45.4)
Fair or poor	56/71 (78.9)	51/68 (75.0)	34/69 (49.3)
* P* value	<.001	<.001	.53
Polypharmacy (≥5 prescription medications)			
No	116/186 (62.4)	74/174 (42.5)	81/182 (44.5)
Yes	60/75 (80.0)	55/73 (75.3)	36/75 (48.0)
* P* value	.01	<.001	.68
Use of sleep medications			
No	137/217 (63.1)	97/200 (48.5)	93/210 (44.3)
Yes	50/71 (70.4)	41/69 (59.4)	33/71 (46.5)
* P* value	.32	.13	.78
Trauma			
No	50/88 (56.8)	35/85 (41.2)	33/85 (38.8)
Yes	126/181 (69.6)	95/168 (56.5)	89/179 (49.7)
* P* value	.04	.02	.11
Anxiety or depression			
No	86/167 (51.5)	74/157 (47.1)	74/163 (45.4)
Yes	90/100 (90.0)	50/93 (53.8)	46/97 (47.4)
* P* value	<.001	.36	.80
Moderate to high stress			
No	49/104 (47.1)	48/98 (49.0)	42/100 (42.0)
Yes	131/172 (76.2)	86/162 (53.1)	80/169 (47.3)
* P* value	<.001	.53	.45

Abbreviations: NA, not available; OSA, obstructive sleep apnea.

^a^ Percentages indicate the proportion exhibiting each sleep deficiency among those with the given participant characteristic, with available case analysis used for each. Proportions representing less than 5 people were suppressed to protect participant anonymity, indicated by NA. Scores of 10 or higher on the insomnia severity index were used to indicate insomnia. Scores of 3 or higher on the snoring, tiredness, observed apnea, pressure, body mass index greater than 35, age greater than 50 years, neck circumference greater than 16 in or 40 cm, and gender questionnaire were used to indicate an elevated risk of obstructive sleep apnea. Insufficient sleep was defined as mean weekday sleep less than 7 hours per night or mean weekend sleep less than 7 hours per night. For each participant characteristic, differences in the prevalence of each sleep deficiency across strata were examined using Fisher exact tests. Demographics were participant self-report.

^b^ The racial category of “other” includes participants with any of the following responses: American Arab, Middle Eastern, and North African; Asian; Native American or Pacific Islander; American Indian or Alaska Native; biracial or multiracial, or not listed.

^c^ Calculated as weight in kilograms divided by height in meters squared.

## Discussion

Despite including a federally designated health disparity population with well-documented insufficient sleep hotspots, rural Appalachian adults remain critically understudied.^3,4,5^ This study addresses knowledge gaps by documenting remarkably high rates of sleep deficiencies; insomnia affected 64.9% of participants (vs approximately 10% nationally), OSA risk 51.3% (vs approximately 38% nationally), and insufficient sleep 44.8% (vs approximately 35% nationally),^7^ revealing a substantially disproportionate burden of sleep disorders in this population. The pronounced sociodemographic associations—particularly the steep socioeconomic gradient in insomnia, declining from 82.9% among the lowest earners to 44.4% among the highest—underscore how social determinants fundamentally shape sleep health in underserved communities. Study limitations include cross-sectional design, self-report measures, female overrepresentation, and required internet access. Ethnic homogeneity reflects regional demographics but limits generalizability. The identification of distinct risk profiles across sleep conditions (sex differences, employment status, living arrangements, and health behaviors) demonstrates that sleep deficiencies require targeted, multifaceted interventions rather than one-size-fits-all approaches. These findings establish the foundation for developing culturally appropriate, equity-focused sleep health interventions in rural Appalachian populations.

## Notes

Supplement 1. eAppendix. eReferences.

Supplement 2. Data Sharing Statement
