# Supplementary material for: Social Determinants of Sleep Health Inequities Among Rural Appalachian Adults
Source: JAMA Netw Open. 2026 Apr 9;9(4):e265908. doi: 10.1001/jamanetworkopen.2026.5908 (PMC13067004; doi:10.1001/jamanetworkopen.2026.5908)
Supplement: Supplement 1. — eAppendix. eReferences. [file jamanetwopen-e265908-s001.pdf]

Supplemental Online Content

Moloney ME, Slade E, Chung J, Mitu MM, Grandner MA, Moga DC. Social determinants of sleep health inequities among rural Appalachian adults. *JAMA Netw Open*. 2026;9(4):e265908. doi:10.1001/jamanetworkopen.2026.5908

eAppendix.

eReferences.

This supplemental material has been provided by the authors to give readers additional information about their work.

© 2026 [First author last name and initials or group name] et al. [Journal name].

eAppendix.

Measures

Insomnia Severity

Insomnia severity was assessed using the Insomnia Severity Index (ISI).[1] The ISI indexes two-week insomnia impact and severity using 7 items, with total scores ranging from 0–28. ISI scores ≥10 were used to indicate insomnia, a threshold found optimal for indicating clinically significant insomnia in a community sample.[2]

Obstructive Sleep Apnea

OSA risk was assessed using the STOP-Bang questionnaire, an 8-item screening tool that evaluates snoring, tiredness, observed apneas, blood pressure, body mass index, age, neck circumference, and gender.[3] Each item receives a score of 0 or 1, with total scores ranging from 0–8. We defined "high OSA risk" as a STOP-Bang score ≥3, which is the standard clinical cutoff with demonstrated sensitivity of 93% and specificity of 43% for moderate-to-severe OSA and is widely used in primary care and research settings for identifying individuals who warrant further sleep evaluation.[4]

Insufficient Sleep

Sleep duration was assessed using items from the Self-Assessment of Sleep Survey Split (SASS-Y) instrument.[5] We computed sleep duration as the difference between participants' "fell asleep time" (reported bedtime plus sleep onset latency) and wake time, minus any reported time spent awake during the night. Insufficient sleep was defined as average weekday sleep <7 hours per night or average weekend sleep <7 hours per night, based on the American Academy of Sleep Medicine and Sleep Research Society recommendation of at least 7 hours of sleep per night for adults.[6]

Demographic Information

Demographics information was collected from participants including age, sex, race, ethnicity, education, employment status, income, and living status (alone vs. with others).

Health, Health Behaviors, and Functioning

© 2026 [First author last name and initials or group name] et al. [Journal name].

Alcohol use was assessed via self-report using a substance use history questionnaire for the prior 90 days. Heavy drinking for men was defined as consuming ≥5 drinks within a 2-hour period on any day, or ≥15 drinks per week. Heavy drinking for women was defined as consuming ≥4 drinks during a 2-hour period on any day, or ≥8 drinks per week. Cigarette use was assessed via self-report of cigarette use in the past month.

Diet quality was assessed using the Rapid Eating Assessment for Patients-Shortened Version (REAP-S).[7] Participants reported their food intake during the previous week across 13 items. Responses were scored as follows: "usually/often" received 1 point, "sometimes" received 2 points, and "rarely/never or does not apply to me" received 3 points. Total scores ranged from 13 to 39, with higher scores indicating better diet quality.

Physical activity was measured using the International Physical Activity Questionnaire (IPAQ).[8] Moderate to vigorous physical activity (MVPA) was determined from yes/no responses to questions about participation in vigorous physical activities (such as heavy lifting, digging, aerobics, or fast bicycling) or moderate physical activities (such as carrying light loads, bicycling at regular pace, or doubles tennis, excluding walking) during the past 7 days.

Social support was defined using 8 items from the modified Medical Outcomes Study Social Support Survey (mMOS-SS).[9] Each item ranged from 1 to 5. The social support score was calculated as the average of any observed items (ignoring missing items), then transformed to a 0-100 scale with higher scores indicating more support.[10]

Body mass index (BMI) was calculated from self-reported height and weight.[11] Overall health status was assessed using a single item from the Short Form-20 Health Survey (SF-20): "In general, would you say your health is:" with response options of excellent, very good, good, fair, or poor.[12] Fair and poor were combined to avoid small cell counts and protect participant anonymity.

<u>Medication Use</u>

Participants self-reported their medication use. We assessed polypharmacy ([5+ daily prescription medications] yes/no) and use of sleep medications ([prescription or over-the-counter] yes/ no).

<u>Trauma Exposure</u>

© 2026 [First author last name and initials or group name] et al. [Journal name].

Trauma exposure (yes/no) was assessed using the Brief Trauma Questionnaire.[13] This validated 10-item instrument assesses lifetime exposure to potentially traumatic events including accidents, natural disasters, physical or sexual assault, combat exposure, and other life-threatening experiences.

Depression and Anxiety Symptoms

We measured depressive symptomatology using the Patient Health Questionnaire (PHQ)-9[14] which asks respondents to rate how often they have been bothered by various depressive symptoms during the last two weeks on a 4-point Likert scale (0=Not at all, 1=Several days, 2=More than half the days, 3=Nearly every day). Total scores range from 0 to 27, with higher scores indicating greater depressive symptoms. Participants missing any of the 9 items on the PHQ-9 were considered to have the total depressive symptoms variable missing.

Anxiety symptoms were measured by the Generalized Anxiety Disorder- 7 (GAD-7)[15] comprised of 7 questions asking about anxiety symptoms experienced over the past two weeks with the same 4 point scale. Total scores range from 0-21 with higher scores suggesting more severe anxiety symptoms. Participants missing any of the 7 items on this scale were considered to have the total anxiety symptoms variable missing.

Considering the highly comorbid nature of anxiety and depression in our sample, we created a single binary variable for analysis representing moderate to severe anxiety or depression symptoms, defined as participants scoring ≥10 on either the PHQ-9 (moderate to severe depression) or GAD-7 (moderate to severe anxiety).

Perceived Stress

Stress was measured using the 10-item Perceived Stress Scale (PSS).[16] The scale asks respondents to rate how often they have experienced specific thoughts and feelings during the past month on a 5-point Likert scale ranging from 0 ("never") to 4 ("very often"). Total scores range from 0 to 40. Higher scores indicate greater psychological distress. The numerical total score of ≥14, indicating moderate to high stress, was used for examining associations with sleep outcomes.[17] Participants missing any of the 10 items on the PSS were considered to have the total perceived stress score missing.

© 2026 [First author last name and initials or group name] et al. [Journal name].

eReferences

1. Bastien CH, Vallières A, Morin CM. Validation of the Insomnia Severity Index as an outcome measure for insomnia research. *Sleep Med*. Jul 2001;2(4):297-307. doi:10.1016/s1389-9457(00)00065-4

2. Morin CM, Belleville G, Bélanger L, Ivers H. The Insomnia Severity Index: psychometric indicators to detect insomnia cases and evaluate treatment response. *Sleep*. May 01 2011;34(5):601-8. doi:10.1093/sleep/34.5.601

3. Chung F, Yegneswaran B, Liao P, et al. STOP questionnaire: a tool to screen patients for obstructive sleep apnea. *Anesthesiology*. May 2008;108(5):812-21. doi:10.1097/ALN.0b013e31816d83e4

4. Nagappa M, Liao P, Wong J, et al. Validation of the STOP-Bang Questionnaire as a Screening Tool for Obstructive Sleep Apnea among Different Populations: A Systematic Review and Meta-Analysis. *Plos One*. Dec 14 2015;10(12)doi:10.1371/journal.pone.0143697

5. Dietch JR, Sethi K, Slavish DC, Taylor DJ. Validity of Two Retrospective Questionnaire Versions of the Consensus Sleep Diary: The Whole Week and Split Week Self-Assessment of Sleep Surveys. *Sleep Medicine*. Dec 2019;64:S92-S92.

6. Watson NF, Badr MS, Belenky G, et al. Joint Consensus Statement of the American Academy of Sleep Medicine and Sleep Research Society on the Recommended Amount of Sleep for a Healthy Adult: Methodology and Discussion. *Sleep*. Aug 1 2015;38(8):1161-1183. doi:10.5665/sleep.4886

7. Segal-Isaacson CJ, Wylie-Rosett J, Gans KM. Validation of a short dietary assessment questionnaire: The Rapid Eating and Activity Assessment for Participants Short Version (REAP-S). *Diabetes Educator*. Sep-Oct 2004;30(5):774-781. doi:Doi 10.1177/014572170403000512

8. Craig CL, Marshall AL, Sjöström M, et al. International physical activity questionnaire: 12-country reliability and validity. *Med Sci Sport Exer*. Aug 2003;35(8):1381-1395. doi:10.1249/01.Mss.0000078924.61453.Fb

9. Moser A, Stuck AE, Silliman RA, Ganz PA, Clough-Gorr KM. The eight-item modified Medical Outcomes Study Social Support Survey: psychometric evaluation showed excellent performance. *Journal of Clinical Epidemiology*. Oct 2012;65(10):1107-16. doi:10.1016/j.jclinepi.2012.04.007

10. Sherbourne CD, Stewart AL. The MOS social support survey. *Social Science & Medicine*. 1991;32(6):705-14. doi:10.1016/0277-9536(91)90150-b

11. Zierle-Ghosh A, Jan A. Physiology, Body Mass Index. *StatPearls*. StatsPearls Publishing; 2023. Accessed September 5, 2025. https://www.ncbi.nlm.nih.gov/books/NBK535456/

12. Stewart AL, Hays RD, Ware JE. The Mos Short-Form General Health Survey - Reliability and Validity in a Patient Population. *Medical Care*. Jul 1988;26(7):724-732. doi:Doi 10.1097/00005650-198807000-00007

13. Kubzansky LD, Bordelois P, Jun HJ, et al. The Weight of Traumatic Stress A Prospective Study of Posttraumatic Stress Disorder Symptoms and Weight Status in Women. *Jama Psychiatry*. Jan 2014;71(1):44-51. doi:10.1001/jamapsychiatry.2013.2798

14. Kroenke K, Spitzer RL, Williams JBW. The PHQ-9 - Validity of a brief depression severity measure. *Journal of General Internal Medicine*. Sep 2001;16(9):606-613. doi:DOI 10.1046/j.1525-1497.2001.016009606.x

15. Spitzer RL, Kroenke K, Williams JB, Lowe B. A brief measure for assessing generalized anxiety disorder: the GAD-7. *Arch Intern Med*. May 22 2006;166(10):1092-7. doi:10.1001/archinte.166.10.1092

© 2026 [First author last name and initials or group name] et al. [Journal name].

16. Cohen S, Kamarck T, Mermelstein R. A global measure of perceived stress. *J Health Soc Behav*. Dec 1983;24(4):385-96.
17. Cohen S, Janicki-Deverts D. Who's stressed? Distributions of psychological stress in the United States in Probability Samples from 1983, 2006, and 2009. *Journal of Applied Social Psychology*. 2012;42:1320-1334.

© 2026 [First author last name and initials or group name] et al. [Journal name].
