# Supplementary material for: Social Determinants of Sleep Health Inequities Among Rural Appalachian Adults
Source: JAMA Netw Open. 2026 Apr 9;9(4):e265908. doi: 10.1001/jamanetworkopen.2026.5908 (PMC13067004; doi:10.1001/jamanetworkopen.2026.5908)
Supplement: Supplement 2. — Data Sharing Statement [file jamanetwopen-e265908-s002.pdf]

Data Sharing Statement

Moloney. Social Determinants of Sleep Health Inequities Among Rural Appalachian Adults. *JAMA Netw Open*. Published April 09, 2026. doi:10.1001/jamanetworkopen.2026.5908

Data
Data available: Yes
Data types: Deidentified participant data
How to access data: The data that support the findings of this study are available on request from the corresponding author (memoloney@miami.edu). The data are not publicly available to protect the confidentiality of participants from this small, geographically identifiable rural community. Deidentified individual participant data may be shared with qualified researchers following approval of a methodologically sound proposal and completion of a data use agreement. Requests should be directed to Dr. Mairead Moloney at memoloney@miami.edu
When available: With publication

Supporting Documents
Document types: None

Additional Information
Who can access the data: Deidentified individual participant data may be shared with qualified researchers following approval of a methodologically sound proposal and completion of a data use agreement.
Types of analyses: Deidentified individual participant data may be shared with qualified researchers following approval of a methodologically sound proposal and completion of a data use agreement.
Mechanisms of data availability: Deidentified individual participant data may be shared with qualified researchers following approval of a methodologically sound proposal and completion of a data use agreement.
